# Baseline characteristics of patients with degenerative cervical myelopathy in Australia: analysis from the MYelopathy NAtural History registry

**DOI:** 10.1111/ans.19354

**Published:** 2024-12-17

**Authors:** Nashwa Najib, Alper Yataganbaba, Nancy E. Briggs, Ali Ghahreman, Ralph Mobbs, Prashant J. Rao, Mitchell Hansen, Mark Davies, Saeed Kohan, Vinay Kulkarni, Alisha Sial, Anuruthran Ambikaipalan, Ashish D. Diwan

**Affiliations:** ^1^ University of New South Wales (UNSW) Sydney Australia; ^2^ Royal Melbourne Hospital Melbourne Australia; ^3^ St George Private Hospital Sydney Australia; ^4^ Neurospine Clinic Sydney Australia; ^5^ Brain and Spine Surgery Sydney Australia; ^6^ Newcastle Brain and Spine Newcastle Australia; ^7^ Epworth Hospital Melbourne Australia

**Keywords:** degenerative cervical myelopathy, longitudinal cohort, observational study, patient registry, prognosis, study protocol

## Abstract

**Background:**

Degenerative Cervical Myelopathy (DCM) is the most common cause of non‐traumatic, chronic spinal cord dysfunction worldwide, causing debilitating disability with a diminishing quality of life. The natural history of DCM is poorly understood. This is a preliminary report of the first 60 patients recruited to the MYelopathy NAtural History (MYNAH) Registry.

**Methods:**

MYNAH Registry is an investigator‐initiated, multicenter, prospective, non‐interventional, longitudinal, national observational study (Registry ID ACSQHC‐ARCR‐258). Given the observational nature of the Registry, participants' clinical management plan is neither changed nor affected. Participants are recruited via an opt‐in approach. A patient with DCM diagnosed by a spine/neurosurgeon after 1st January 2018 onwards is eligible to participate regardless of their surgical status. The Patient‐Reported Outcome Measures (PROMs) are NDI, EQ5D5L and EQ‐VAS; and the Practitioner‐Reported Outcome Measures (PrROMs) are mJOA Score and Nurick Grade.

**Results:**

Sixty participants (*n* = 60) have now been recruited of which male participants 34 (56.7%) and females are 26 (43.3%), with a mean age of 62.3 years (SD 14.1) and biospecimens for Proteomics have been collected from 33 (66%) participants. The median mJOA Score was 16.5 (8–18), with myelopathy severity recorded as mild in 42 (70%), moderate in 13 (21.7%) and severe in 5 (8.3%) participants. Median Nurick Grade 0 (0–5), NDI 14 (0–45), EQ5D5L Score 0.850 (−0.288–1) and EQ‐VAS 70 (10–96).

**Conclusions:**

The MYNAH National DCM Registry in Australia is a novel spinal surgical initiative, that will inform the decision(s) to proceed with the scientific, evidence‐based and personalised management of DCM globally in the future.

## Graphical abstract

The MYNAH Registry is a multicenter, prospective, non‐interventional, observational study to understand the natural history of DCM supplemented with longitudinal follow‐ups and proteomics discovery.

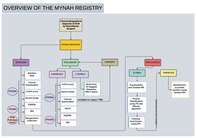

## Introduction

Degenerative Cervical Myelopathy (DCM) is the most prevalent cause of chronic, non‐traumatic spinal cord dysfunction worldwide, resulting in major disability and the lowest quality of life among all chronic diseases.^1^ DCM encompasses a spectrum of clinical features, including localised neck pain, progressive motor weakness, gait abnormalities (difficulty with tandem gait), paraesthesia, diminished sensory perception, compromised fine motor skills, potential bowel, bladder and sexual dysfunction, persistent cervical discomfort, increased muscle tone or spasticity and alterations of reflex responses. It can lead to paralysis and even death if treatment is not sought.^1^ The degree of neurological impairment can be measured with a modified Japanese Orthopaedic Association (mJOA) Score or Nurick Grade. It has also been found that DCM patients suffer the worst quality of life among all chronic diseases.^2^ Although, surgical spinal cord decompression remains the mainstay of treatment for DCM patients,^3^ very few patients achieve complete recovery.^4^ Consequently, most patients with DCM confront a life‐long disability.

The prevalence of DCM has been estimated to be 1.6 to 4.04 per 100 000 people and accounts for 22% of all non‐traumatic spinal cord injuries in Australia. It is expected to increase as the global population ages.^5^ Spine‐related problems contribute largely to illness, pain and disability in the Australian population. In 2022, the Australian Burden of Disease Study reported that spinal problems are the third leading cause of disease, accounting for 4% of Australia's total disease burden (AIHW 2022). Despite its prevalence and disease burden, DCM remains under‐recognised.

Existing evidence suggests that research on DCM remains inefficient,^6^ with the main focus on evaluating surgical techniques and post‐op outcomes of DCM patients. However, the natural history of DCM is not a prevalent research theme. This hinders the ability of clinicians to offer adequate counselling and treatment options to DCM patients. Furthermore, since not all patients with DCM are managed by surgery, there is a gap in knowledge in understanding the disease outcomes for patients who do not undergo surgery. Since DCM is a neglected research topic, the AO Spine REsearch objectives and COmmon Data Elements for Degenerative Cervical Myelopathy (RECODE DCM) have prioritized research in DCM encouraging much‐needed research.^7^

To address the RECODE DCM priorities the MYelopathy NAtural History (MYNAH) Registry was established in December 2022 to address the RECODE DCM research priorities of Raising Awareness, Understanding Natural History, Defining Diagnostic Criteria, Assessment and Monitoring, and Socioeconomic impact by evaluating the clinical and quality of life outcomes of DCM patients in the Australian community. The MYNAH Registry (Registry ID: ACSQHC‐ARCR‐258) is a Clinical Quality Registry (CQR) listed on the Australian Register of Clinical Registries (the Register) and published on the Australian Commission on Safety and Quality in Health Care (the Commission) website (https://www.safetyandquality.gov.au/publications‐and‐resources/australian‐register‐clinical‐registries) which is the authority that maintains a record of all the clinical registries existing in Australia and publishes them on the Register to facilitate collaboration and awareness of registries in Australia.

The MYNAH Registry provides an ideal National platform to collect longitudinal data from DCM patients (operated and non‐operated) and monitor their outcomes through bi‐annual follow‐ups, offering a comprehensive understanding of the natural history of DCM.

### Study objectives

The overall objective is to describe the natural history of DCM; nested within that, to compare the outcomes of DCM in operated and non‐operated patients, to describe the signalling pathways and identify biomarkers associated with DCM using Global and Targeted Proteomics.

## Methods

### Participant description and recruitment

Both male and female patients are recruited in the study and should meet the indicated inclusion and exclusion criteria. The inclusion criteria are (i) All patients with DCM diagnosed by specialist spine surgeons and neurosurgeons from participating study sites from 1st January 2018 onwards. Cases will include patients for whom surgery was offered or performed (regardless of the type of surgery) as well as those for whom surgery was not indicated, not performed or patient opted against surgery; (ii) Patients recorded with ICD‐10 Codes: M50.0+, M50.1, M50.3, M47.1, G99.2 in South Eastern Sydney Local Health District (SESLHD) Electronic Medical Record (EMR) databases; (iii) Patients who provide informed consent. The exclusion criteria are (i) Patients with a cognitive decline or intellectual disability and (ii) Patients who are unable to or unwilling to provide informed consent.

**Fig. 1:**
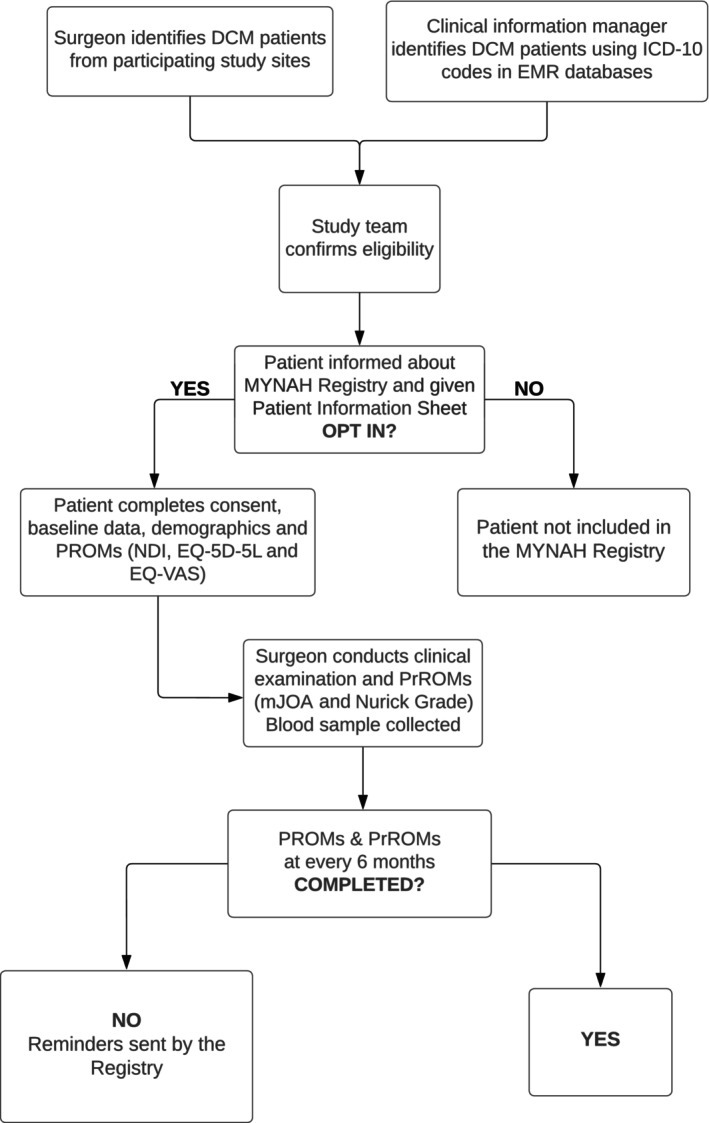
MYNAH Registry Process. EQ‐5D‐5L: EuroQol EQ‐5D is a standardized measure of health status developed by the EuroQol Group in order to provide a simple, generic measure of health for clinical and economic appraisal. 5D represents five dimensions; 5L represents five levels; DCM: Degenerative Cervical Myelopathy; EMR: Electronic Medical Record; mJOA Score: modified Japanese Orthopaedic Association Score; MYNAH: MYelopathy NAtural History; NDI: Neck Disability Index; PROMs: Patient‐Reported Outcome Measures; PrROMs, Practitioner‐Reported Outcome Measures

Participants are recruited using an opt‐in approach. The Research Team contacts eligible participants, to inform them about the MYNAH Registry and provide a patient information sheet. Potential participants are either sent a link to REDCap via e‐mail or are sent paper forms to opt‐in and complete consent along with three Patient‐Reported Outcome Measures (PROMs) namely NDI, EQ5D5L and EQ‐VAS and visit their respective study site for neurological examination, blood collection and completion of two Practitioner‐Reported Outcome Measures (PrROMs) namely mJOA Score and Nurick Grade. The MYNAH Registry Process is described in Figure 1.

### Ethics

Ethical approval was obtained from the University of New South Wales (UNSW) Human Research Ethics Committee (HREC) Executive on 25th November 2022 (iRECS3634). Data custodian approval from SESLHD to access EMR data using ICD‐10 codes was obtained on 9th December 2022 (Ref No. T22/84228). The Registry was developed according to the Framework for Australian Clinical Quality Registries as recommended by the Australian Commission on Safety and Quality in Health Care.^8^

### Description of the MYNAH Registry and Study Protocol

The MYNAH Registry is an investigator‐initiated, multicentre, prospective, observational cohort study enrolling patients with DCM across Australia. Patients are managed according to their regular treating physicians, surgeons or general practitioners (GPs) without interference from the Research Team. Potential participants give informed consent to participate in the registry and data collection begins which includes baseline data collection and biannual (6‐monthly) follow‐ups. At baseline, comprehensive data is collected which includes demographics, clinical examination, mJOA Score, Nurick Grade, baseline data, MRI of cervical spine and biospecimen collection. At each 6‐monthly follow‐up, in addition to PROMs and PrROMs, participants' cognition is assessed using the Montreal Cognitive Assessment (MoCA). MoCA Blind/Telephone versions 8.1, 8.2 and 8.3 are alternatively administered at each follow‐up to minimise practice effects due to re‐exposure to the same MoCA items. A Modified Monash Model (MMM) was used for remoteness classification which categorizes participants from MM 1 (major city) to MM 7 (very remote) based on their postcodes.

MYNAH Registry was created using the Research Electronic Data Capture (REDCap) cloud database. A longitudinal project design was opted to create the REDCap database for the MYNAH Registry. Longitudinal data collection was used with defined events. In the development status of the MYNAH Registry project, data collection instruments and surveys were designed using the online designer feature on REDCap. The instruments created were Case Report Form (CRF), e‐consent, demographics, baseline data, NDI, EQ5D5L, EQ‐VAS, mJOA Score, Nurick Grade, clinical examination, MRI, biospecimen collection, follow‐up questionnaire and MoCA. The MYNAH Registry was registered with EuroQol to facilitate access and enable non‐commercial use of EQ5D5L and EQ‐VAS. Events were defined, and instruments were designated for each event. Only arm 1 was used and events were defined with event labels as baseline, first follow‐up, second follow‐up, third follow‐up, fourth follow‐up and so on. All the instruments were designated at the baseline event. NDI, EQ5D5L, EQ‐VAS, mJOA Score, Nurick Grade, clinical examination, follow‐up questionnaire and MoCA were designated for the remaining follow‐up events. User rights and permissions were granted to the MYNAH Registry team. The project was thoroughly tested in three rounds before moving the MYNAH Registry to production status. A test participant list was created, and surveys were tested. Test records were created to test data collection instruments, calculations, and survey completion emails. Data were tested by creating reports and exporting data to Excel and R Studio.

### Follow‐up

Follow‐ups for all participants are conducted every 6 months. Participant completes four PROMs (NDI, EQ5D5L, EQ‐VAS and follow‐up questionnaire). The participant visits their study site for neurological examination, completion of PrROMs (mJOA Score and Nurick Grade) and MoCA by the practitioner. Non‐operated participants undergo an MRI of the cervical spine once every year as a standard of care practice.

If the participant chooses to withdraw consent at any time during the study, the participant is excluded from the MYNAH Registry. If the participant does not withdraw their consent and does not wish to continue to participate, they are then marked as being lost to follow‐up.

### Data collection

Data collection commenced on 1st December 2022. Participant data are collected at baseline and then at every 6‐month follow‐up for up to 2 years and subsequent review will be conducted for future annual or bi‐annual follow‐ups. The data collection schedule is described in Table 1.

**Table 1:** Data collection schedule

Assessment parameters	Baseline visit	6 months follow‐up
Consent	**●**	
Baseline data	**●**	
Demographics	**●**	
Clinical examination	**●**	**●**
mJOA Score	**●**	**●**
Nurick Grade	**●**	**●**
NDI	**●**	**●**
EQ‐5D‐5L	**●**	**●**
EQ‐VAS	**●**	**●**
Follow‐up questionnaire		**●**
MoCA		**●**
MRI cervical spine†	**●**	
Blood collection	**●**	

† MRI cervical spine: existing MRI acceptable at baseline. New MRI at every 1‐year follow‐up for non‐operated patients. EQ‐5D‐5L: EuroQol EQ‐5D is a standardized measure of health status developed by the EuroQol Group in order to provide a simple, generic measure of health for clinical and economic appraisal. 5D represents five dimensions; 5L represents five levels; mJOA Score: Modified Japanese Orthopaedic Association Score; MoCA: Montreal Cognitive Assessment; NDI: Neck Disability Index.

### Governance, data safety, monitoring and quality control

The MYNAH Registry is maintained on the REDCap cloud server. All participant data is stored in a deidentified format on the REDCap server, which is highly secure having restricted access and is overlooked by the Data Custodian (AD). All biospecimens are stored in a deidentified format at −80°C in freezers located in Spine Labs which is a restricted facility with card access only. Access to REDCap server and Spine Labs is granted exclusively to the authorised MYNAH Research Team ensuring complete data security and data confidentiality compliance.

The MYNAH REDCap database incorporates mandatory fields and if left blank will not enable the user to submit the form and will be flagged as incomplete. The MYNAH Registry's Record Status Dashboard is checked regularly by a member of the Research Team and flagged forms are followed up by sending reminder emails and calls to the clinicians and participants on two different occasions with a gap of 1 week, following which they are marked as a loss to follow‐up. Discrepancies that cannot be solved are flagged and discussed at monthly data review meetings. Participants are provided with the choice to opt out of the study if they no longer wish to continue participating in the MYNAH Registry. This ensures timely resolution of queries and complete data collection for each participant.

Database governance procedures, data management and access, compliance with ethical obligations and protection of patient data privacy and confidentiality are per the Guidelines issued by the National Health and Medical Research Council (NHMRC)^9^ and the Guidelines stated under Section 95^10^ and Section 95A^11^ of the Privacy Act 1988 issued by the Federal Register of Legislation, Australian Government.

### Statistical and bioanalytical plan

The minimum data set includes baseline data, demographics, clinical examination, NDI, EQ5D5L, EQ‐VAS, mJOA Score, Nurick Grade, MoCA and follow‐up questionnaire. Initial data analysis will focus on descriptive statistics to provide summary information on participant characteristics and demographics. Assessment of feasibility will be performed to provide performance metrics such as cumulative recruitment of participating sites, response rate and response time for Practitioner‐Reported Outcomes (PrROs) and Patient‐Reported Outcomes (PROs). Outcome measures will be analysed using the Generalized Linear Mixed Model (GLMM) and the Censored Normal Regression Model (CNRM). Proteins will be identified using the Proteome Discoverer software (Thermo Fisher Scientific; Waltham, Massachusetts, USA, V3.1) and using Mascot algorithms for protein identification within the UniProt database (downloaded January 2023). Pathway enrichment will be assessed using Ingenuity Pathway Analysis (IPA) (Qiagen; Redwood City, USA). Analyses will be performed using R V4.2.2 and SAS V9.4.

## Results

Participant recruitment is ongoing from 14 approved study sites across Australia (Table 2). There were 60 participants (mean (SD) age; 62.3 (14.1) years and 34 (56.7%) males) and biospecimens for Proteomics have been collected from 33 (66%) participants since the recruitment of the index patient in December 2022. The majority were married 37 (61.7%) and Australian 35 (58.3%) followed by British 6 (10.0%), Chinese 3 (5.0%), Indigenous Australian 2 (3.3%) and other ethnicities 14 (23.8). A tertiary level of education was attained by 29 (48.3%), 25 (41.7%) were retired and 3 (5.0%) were disabled. The most commonly used pain medication was Paracetamol 26 (43.3%) followed by Opioids 10 (16.7%), prescribed anti‐inflammatory 8 (13.3%), neuromodulators 7 (11.7%), OTC anti‐inflammatory 6 (10.0%) and benzodiazepine 3 (5.0%). The most commonly reported comorbidity was Hypertension 15 (25.0%) followed by DM 12 (20.0%), Asthma 11 (18.3%), Cancer 7 (11.7%) and Rheumatoid arthritis 5 (8.3%). Cancer 22 (36.7%) was the most common family history reported followed by Hypertension 13 (21.7%), DM 13 (21.7%), CAD 13 (21.7%), Stroke 13 (21.7%) and DCM 4 (6.7%). Most participants were on Physiotherapy 21 (35.0%). Alcohol was consumed by 37 (61.7%) and 5 (8.3%) had a positive smoking status. Forty‐three (72%) of the participants belonged to Metropolitan areas (MM 1) while the remaining 17 (28%) belonged to MM 2 – MM 7. According to mJOA Score, 42 (70.0%) participants had mild myelopathy, 13 (21.7%) had moderate myelopathy and 5 (8.3%) had severe myelopathy with 24 (40%) having had previous cervical spine surgery.

**Table 2:** MYNAH Study Sites

S. No.	Study site	Location	State
1	Dr AD	St George Private Hospital, Kogarah	NSW
2	Dr MD	St George Private Hospital, Kogarah	NSW
3	Dr SK	St George Private Hospital, Kogarah	NSW
4	Dr AK	St George Private Hospital, Kogarah	NSW
5	Dr BS	NSW Spine Specialists, Norwest	NSW
6	Dr BH	NSW Spine Specialists, Norwest	NSW
7	Dr PR	Norwest Private Hospital, Bella Vista	NSW
8	Dr MH	Newcastle Brain and Spine, New Lambton Heights	NSW
9	Dr RM	Neuro Spine Clinic, POWH, Randwick	NSW
10	Dr RY	CNC Victoria, Melbourne	Victoria
11	Dr AA	Epworth Healthcare, Melbourne	Victoria
12	Dr YY	Spine Surgery, Melbourne	Victoria
13	Dr AK	Westmead Hospital	NSW
14	SESLHD EMR	South Eastern Sydney Local Health District Network	NSW

## Discussion

Clinical registries are databases that systematically collect patient health information in a temporal pattern. The vital role of clinical registries in health care dates back to 1856 when Ove Guldberg Hoegh established the world's first national patient registry; the National Leprosy Register in Norway.^12^ Establishing clinical registries helps to understand the natural history of a disease, the safety and efficacy of treatments and procedures, monitor outcomes and provide reports on the quality of care. Registries provide benchmarks for clinical performance and provide evidence‐based good clinical practice. Clinical registries improve clinical practice and overall health outcomes. There are various degenerative disease registries in Australia such as the Australian Parkinson's Disease Registry, The Australia Dementia Network Clinical Quality Registry, the Australian Multiple Sclerosis AHSCT Registry and the MiNDAUS National MND/ALS Registry.

The MYNAH Registry is Australia's first clinical registry for DCM patients to provide longitudinal, long‐term follow‐ups, assess disease severity, monitor long‐term outcomes, provide quality‐of‐life measures for DCM and provide a Health Technology Assessment (HTA) for DCM. The unique database is the most detailed analysis of the natural history of DCM. The high‐quality, non‐biased data can be used reliably for clinical practice purposes, medical research and health policymaking and evaluation.

The annual cost for maintaining the MYNAH Registry is estimated at AUD$600000. Funding will be sought from philanthropy, Spinal Cord Injuries Australia (SCIA) and corporate investors. Further grants for funding and ethical approval for the multicentre National DCM Registry will be sought from The National Health and Medical Research Council (NHMRC), under the National Approach to Single Ethical Review of Multi‐centre Research (National Certification Scheme) in Australia. NHMRC is the main statutory authority of the Australian Government responsible for medical research and under the National Certification Scheme recognises a single ethical and scientific review of Multi‐centre human research within Australian jurisdictions.^13^ This enables the researchers to submit an ethics application to one Human Research Ethics Committee (HREC) associated with a certified institution instead of multiple HRECs. A single ethics application will be submitted to either University of New South Wales HREC A (EC00397) and B (EC00142) or South Western Sydney Local Health District Human Research Ethics Committee (EC00136) for obtaining approval following which an application for certification will be submitted to the NHMRC HREC. Funding for scale‐up and capacity building of the MYNAH Registry is being sought with Medical Research Future Funds (MRFF) and CQR Capacity Building Grants in Australia. Feasibility outcomes will be measured by analysing the recruitment rate, retention rate, clinician compliance in data collection and participant compliance in completing PROMs. Assessment of feasibility will help identify potential problems impacting study execution. A consensus meeting to discuss the learnings and adaptations from the pilot phase will inform necessary amendments.

The strength of the MYNAH Registry is temporal follow‐up which will help to monitor disease progression and clinical deterioration in DCM participants. MYNAH Registry is an investigator‐initiated observational, non‐interventional, multicentre study. Compared to Randomized Clinical Trials (RCT), observational studies are subject to confounding and may bias the results. There is more risk of systematic error and selection bias. Hence, clinical registries can be challenged by RCTs. Participant loss to follow‐up is a potential limitation of the MYNAH Registry. Another limitation is the opt‐in approach of participant recruitment, which results in a lower response rate.^14^

Participant recruitment and data collection for the MYNAH Registry is ongoing. We would like to take this opportunity to invite spine surgeons and neurosurgeons to contribute their DCM patients to the MYNAH Registry to increase the national coverage of DCM patients in the Registry. The MYNAH Registry is Australia's first patient registry to understand the natural history of DCM; Proteomics holds the potential for understanding the pathways involved and identifying possible biomarkers for DCM. The MYNAH Registry holds scope in understanding the natural history of DCM and contributing to the DCM management guidelines.

## Author contributions

**Nashwa Najib:** Conceptualization; data curation; formal analysis; funding acquisition; investigation; methodology; project administration; visualization; writing – original draft; writing – review and editing. **Alper Yataganbaba:** Writing – review and editing. **Nancy E. Briggs:** Formal analysis. **Ali Ghahreman:** Project administration. **Ralph Mobbs:** Project administration; writing – review and editing. **Prashant J. Rao:** Project administration; writing – review and editing. **Mitchell Hansen:** Project administration. **Mark Davies:** Project administration. **Saeed Kohan:** Project administration. **Vinay Kulkarni:** Data curation. **Alisha Sial:** Project administration. **Anuruthran Ambikaipalan:** Project administration. **Ashish D. Diwan:** Conceptualization; funding acquisition; methodology; project administration; resources; supervision; writing – review and editing.

## Funding information

The MYNAH Registry is partly funded by the South Eastern Sydney Local Health District (SESLHD) RES‐ON Grant Round 2, November 2023. NN is supported by a UIPA scholarship, from the University of New South Wales (UNSW), Sydney, Australia.

## Conflicts of interest

None declared.

## References

[1] Tu J , Vargas Castillo J , Das A , Diwan AD . Degenerative cervical myelopathy: insights into its pathobiology and molecular mechanisms. J. Clin. Med. 2021; 10: 1–2. 10.3390/jcm10061214 PMC8001572 33804008

[2] Oh T , Lafage R , Lafage V *et al*. Comparing quality of life in cervical Spondylotic myelopathy with other chronic debilitating diseases using the short form survey 36‐health survey. World Neurosurg. 2017; 106: 699–706. 28065875 10.1016/j.wneu.2016.12.124

[3] Fehlings MG , Tetreault LA , Riew KD *et al*. A clinical practice guideline for the Management of Patients with degenerative cervical myelopathy: recommendations for patients with mild, moderate, and severe disease and nonmyelopathic patients with evidence of cord compression. Global Spine J. 2017; 7: 70s–83s. 29164035 10.1177/2192568217701914 PMC5684840

[4] Fehlings MG , Ibrahim A , Tetreault L *et al*. A global perspective on the outcomes of surgical decompression in patients with cervical spondylotic myelopathy: results from the prospective multicenter AOSpine international study on 479 patients. Spine (Phila Pa 1976) 2015; 40: 1322–1328. 26020847 10.1097/BRS.0000000000000988

[5] New PW , Farry A , Baxter D , Noonan VK . Prevalence of non‐traumatic spinal cord injury in Victoria, Australia. Spinal Cord 2013; 51: 99–102. 22665222 10.1038/sc.2012.61

[6] Mowforth OD , Davies BM , Goh S , O'Neill CP , Kotter MR . Research inefficiency in degenerative cervical myelopathy: findings of a systematic review on research activity over the past 20 years. Global Spine J 2020; 10: 476–485. 32435569 10.1177/2192568219847439 PMC7222686

[7] Davies BM , Khan DZ , Mowforth OD *et al*. RE‐CODE DCM (REsearch objectives and common data elements for degenerative cervical myelopathy): a consensus process to improve research efficiency in DCM, through establishment of a standardized dataset for clinical research and the definition of the research priorities. Global Spine J. 2019; 9: 65s–76s. 31157148 10.1177/2192568219832855 PMC6512197

[8] National arrangements for clinical quality registries: Australian Commission on Safety and Quality in Health care. [cited 2022 Sep 16]. Available from URL: https://www.safetyandquality.gov.au/our‐work/health‐and‐human‐research/national‐arrangements‐clinical‐quality‐registries.

[9] NHMRC . Guidelines for clinical practice, public health, environmental health and ethics. [cited 2022 Dec 20]. Available from URL: https://www.nhmrc.gov.au/guidelines.

[10] PrivacyAct . Federal Register of Legislation, Australian Government; [cited 2022 Sep 3]. Available from URL: https://www.legislation.gov.au/Details/F2014L01500.

[11] PrivacyAct95A . Federal Register of Legislation, Australian Government; [cited 2022 Sep 3]. Available from URL: https://www.legislation.gov.au/Details/F2014L00243.

[12] Irgens LM , Bjerkedal T . Epidemiology of leprosy in Norway: the history of the National Leprosy Registry of Norway from 1856 until today. Int. J. Epidemiol. 1973; 2: 81–89. 4590337 10.1093/ije/2.1.81

[13] National Certification Scheme for the ethics review of multi‐centre research, National Health and Medical Research Council: Australian Government. [cited 2022 Oct 7]. Available from URL: https://www.nhmrc.gov.au/research‐policy/ethics/national‐certification‐scheme‐ethics‐review‐multi‐centre‐research.

[14] Junghans C , Feder G , Hemingway H , Timmis A , Jones M . Recruiting patients to medical research: double blind randomised trial of "opt‐in" versus "opt‐out" strategies. BMJ 2005; 331: 940. 16157604 10.1136/bmj.38583.625613.AE PMC1261191

